# Secretion-Based Modes of Action of Biocontrol Agents with a Focus on *Pseudozyma aphidis*

**DOI:** 10.3390/plants10020210

**Published:** 2021-01-22

**Authors:** Dhruv Aditya Srivastava, Raviv Harris, Gilli Breuer, Maggie Levy

**Affiliations:** Plant Pathology and Microbiology Department, Robert H. Smith Faculty of Agriculture, Food and Environment, the Hebrew University of Jerusalem, Rehovot 76100, Israel; dhruv.srivastava@mail.huji.ac.il (D.A.S.); raviv_harris@yahoo.com (R.H.); gilli.breuer@mail.huji.ac.il (G.B.)

**Keywords:** biocontrol, epiphytic fungi, secretion, secretome

## Abstract

Plant pathogens challenge our efforts to maximize crop production due to their ability to rapidly develop resistance to pesticides. Fungal biocontrol agents have become an important alternative to chemical fungicides, due to environmental concerns related to the latter. Here we review the complex modes of action of biocontrol agents in general and epiphytic yeasts belonging to the genus *Pseudozyma* specifically and *P. aphidis* in particular. Biocontrol agents act through multiple direct and indirect mechanisms, which are mainly based on their secretions. We discuss the direct modes of action, such as antibiosis, reactive oxygen species-producing, and cell wall-degrading enzyme secretions which can also play a role in mycoparasitism. In addition, we discuss indirect modes of action, such as hyperbiotrophy, induced resistance and growth promotion based on the secretion of effectors and elicitors from the biocontrol agent. Due to their unique characteristics, epiphytic yeasts hold great potential for use as biocontrol agents, which may be more environmentally friendly than conventional pesticides and provide a way to reduce our dependency on fungicides based on increasingly expensive fossil fuels. No less important, the complex mode of action of *Pseudozyma*-based biocontrol agents can also reduce the frequency of resistance developed by pathogens to these agents.

## 1. Introduction

**Figure 1:**
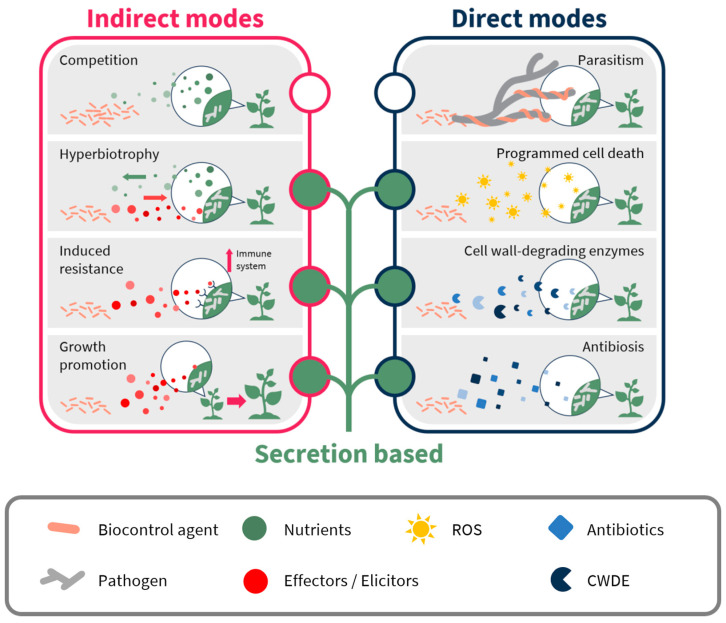
**Biocontrol modes of action.** Illustration of biocontrol secretion-based direct and indirect modes of action against phytopathogens with a focus on *Pseudozyma aphidis*. ROS, reactive oxygen species; CWDE, cell wall-degrading enzymes.

The world population is rapidly growing and is expected to reach over 9 billion people by the year 2050. Feeding such a huge population will require a 70–100% increase in total food production [1]. Since most soils with high productivity potential are already under cultivation, and there is constant shrinkage of cultivated land area due to urban and industrial development [2], it is necessary to maximize agricultural yield. Among the most important contributors to low agricultural yield are pests and pathogens, which are responsible for destroying up to 20% of the world’s harvest, with a further 10% loss post-harvest [3,4]. Fungal pathogens cause most of the diseases in agricultural ecosystems and are responsible for the most costly damage to many crops, including some of the most devastating plant disease epidemics in history [5]. Bacteria can also cause diseases in plants [6]. While relatively less agriculturally and economically damaging than fungi [7], bacteria cause many serious diseases of plants throughout the world, especially in moist and warm environments [6,8]. There are a few ways to reduce these devastating losses. One is to develop resistant crops; however, these need 10 to 15 years to reach the field and can lose their resistance within a decade, with the emergence of new pathogen mutations. Although this method is widely used, it is time-consuming and in general, resistance can only be developed for one crop at a time. The second alternative is the use of fungicides or biomolecules to reduce the losses. Continual use of chemical-based pesticides has allowed farmers to control the spread of pathogens and pests, and has helped ensure stable and prosperous agricultural systems [9,10]. However, continuous pesticide use also results in the widespread problem of pathogen resistance because pathogens tend to find ways to suppress the pesticides’ effects through detoxification methods, such as degradation, or by active efflux of the toxic compound out of their cells using specific transporters [11,12]. In fact, multidrug resistance (MDR) is a major challenge to agriculture the world over [13,14,15,16]. One solution is the use of multiple pesticides with different modes of action in combination or in rotation [17]. However, excessive use of fungicides is deleterious to the ecosystem and human health, and concerns over the potential impact of pesticides on the environment are becoming more pressing. Thus, more stringent pesticide registration procedures and regulations have reduced the number of synthetic pesticides available for agricultural practice. Biological control agents (BCAs) and natural product-based pesticides (biopesticides) are good alternatives to the use of traditional synthetic chemicals as they are less harmful to both the environment and human health [18,19,20,21]. Furthermore, since BCAs can hinder pathogen growth and development, thereby reducing diseases, via a complex combination of direct and indirect modes of action—including antibiosis, mycoparasitism, competition and induced resistance in the host plant—the development of pathogen resistance to BCAs is more complicated [22,23]. As a result, there is growing interest in the exploration and exploitation of naturally occurring microorganisms, such as fungi, for the control of crop diseases [24]. The complex modes of action of BCAs are mainly directed by secreted molecules and proteins that can act as antibiotics, effectors, elicitors and degrading enzymes (Figure 1). This review summarizes the known modes of action of the epiphytic yeast BCAs in general, and *Pseudozyma aphidis* in particular, with a focus on our ability to define the functions of their secretions and possible uses for fungal pathogen control.

## 2. Antibiosis: Fungal Metabolites as a Source for New Pesticides

Many microorganisms, including a large portion of the known BCAs, produce and secrete secondary metabolites with antimicrobial properties, as part of their constant competition with other microorganisms in their natural environment [25] (Figure 1; Table 1). Those metabolites can be exploited as microbial fungicides that may be more environmentally friendly than chemical pesticides, and at the same time lack some of the disadvantages of using living organisms. Due to their natural origins, microbial pesticides are more biodegradable than synthetic chemicals [26]. Microorganisms can synthesize secondary metabolites with versatile chemical structures and diverse biological activities that exceed the scope of synthetic organic chemistry [27]. Therefore, a newly discovered antifungal compound is more likely to have a new and more specific mode of action than the commercial fungicides, and lack cross-resistance to them [28]. The specificity of microbial pesticides is both an advantage and a disadvantage. While it reduces the risk of toxicity to humans, the environment and other beneficial organisms in the field, it also makes it harder for those pesticides to be effectively applied by farmers. Thus, an additional possible use for microbial pesticides could be as lead compounds for the development of novel synthetic fungicides.

Several strains of *Trichoderma* spp. have been found to produce antifungal compounds that are toxic to phytopathogenic fungi such as *Botrytis cinerea*, *Rhizoctonia solani* and *Pythium* spp. [29,30,31,32]. For example, *Trichoderma virens*, *Trichoderma harziarum* and *Trichoderma pseudokoningii* produce unique peptaibols, which are linear peptide antibiotics which can form pores in the membrane and induce programmed cell death (PCD) [31,33,34,35,36]. In addition, the yeast BCAs, such as *Pseudozyma* spp., have been found to secrete antimicrobial compounds. *Pseudozyma rugulosa*, *Pseudozyma flocculosa* and *P. aphidis* all exhibit biological activity against the different powdery mildews with which they are associated [37,38,39,40,41,42,43,44]. While the mechanism of this biological activity has not yet been fully elucidated, *P. rugulosa* and *P. flocculosa* were found to produce unusual extracellular fatty acids with antifungal properties, which cause the release of intracellular ions and proteins when in contact with sensitive fungi [45,46,47,48,49,50]. Furthermore, *Pseudozyma tsukubaensis* and *Pseudozyma prolifica* were shown to produce a very large mycocin with both fungistatic and fungicidal activities. This toxin has very narrow, taxonomically specific activity against some representatives of the orders Microstromatales and Ustilaginales [51,52]. *P. flocculosa* was also found to produce an unusual and rare cellobiose lipid with antifungal activity [53]. This glycolipid, named flocculosin, inhibited several pathogenic fungi, including *Candida albicans* and *Trichosporon asahii* [54]. In a later work, this group reported that flocculosin also has antibacterial properties, directed rather specifically against both aerobic and anaerobic Gram-positive bacteria. In both cases, the antimicrobial activity was achieved by irreversibly damaging the cell membrane of the microorganisms [55]. Another unusual glycolipid, which has a highly similar chemical structure to ustilagic acid, was produced by two other *Pseudozyma* species: *Pseudozyma fusiformata* [56] and *Pseudozyma graminicola* [57]. While the discovery of flocculosin is relatively new, ustilagic acid was discovered and established as an antimicrobial compound more than 60 years ago. Interestingly, the main producer of ustilagic acid is the phytopathogenic fungus *Ustilago maydis* [58]. Recent work has shown that *P. flocculosa* is a close relative of the plant pathogen *U. maydis* whose pathogenicity trait was lost during evolution [59]. However, new evidence indicates that flocculosin plays a secondary, if any, role in the antagonistic activity of *P. flocculosa* against powdery mildews. Instead, the biocontrol process seems to be mediated by a more complex interaction [60].

*P. aphidis* extracts have also been found to inhibit spore germination and linear growth of fungal phytopathogens such as *B. cinerea* and *Alternaria brassicicola* [43], as well as important bacterial phytopathogens such as *Clavibacter michiganensis* subsp. *michiganensis*, *Pseudomonas syringae* pv. *tomato* and *Xanthomonas campestris* pv. *campestris* [61].Those metabolites have not yet been identified but they are largely lipophilic and found to induce reactive oxygen species (ROS) accumulation and PCD in *B. cinerea* hyphal cells [62].

Several commercially used pesticides have been developed from microbial metabolites through chemical modifications, such as fludioxonil, fenpiclonil and strobilurins [26,63,64]. Strobilurins are natural substances isolated mainly from basidiomycetes. The first strobilurin was isolated from liquid culture of *Strobilurus tenacellus*. Natural strobilurins are highly active against yeasts and filamentous fungi, by inhibiting their mitochondrial respiration. Since they break down rapidly in the light, they are not reliable for disease control. Instead, they were used as lead compounds to develop synthetic fungicides that are more stable and more powerful when used commercially in agricultural practice [65]. Several commercial strobilurin-based products are on the market or in development. Since these products are derived from natural products, they are environmentally safe due to their rapid degradation in the environment [64,66]. Since the discovery of the strobilurins, many other microbial metabolites with promising antimicrobial properties have been discovered through activity-based screening against important phytopathogens. Chaetoviridins, which were purified from the broth of *Chaetomium globosum*, exhibit high levels of in vitro and in vivo antifungal activity against *Magnaporthe grisea* and inhibit the development of rice blast disease [67,68]. Fusapyrone and deoxyfusapyrone, isolated from *Fusarium semitectum*, exhibit a broad range of antifungal activity [69]. Several peptides with control efficacy against plant diseases have also been identified. For example, verlamelin is a peptide produced by the fungus *Acremonium strictum* with strong inhibitory activity against powdery mildew [70,71].

## 3. Effectors and Hyperbiotrophy-Dependent Inhibition

**Figure 2:**
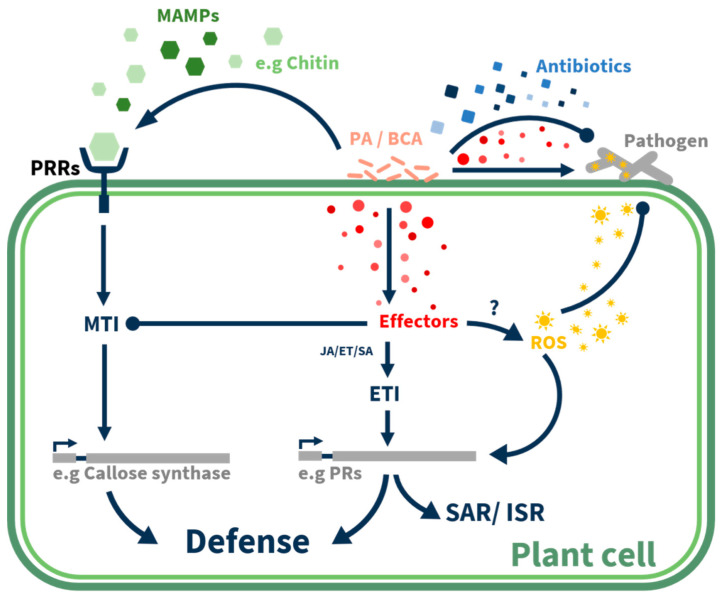
Biocontrol agents’ involvement in plant defense pathways with a focus on *Pseudozyma aphidis*. Recognition of biocontrol agents by microbe associated molecular patterns (MAMPs) and suppression of MAMP-triggered immunity (MTI) by biocontrol agents, while activation the effector-triggered immunity (ETI) and expression of pathogenesis relate genes (PRs), and later on activation of the systemic acquired resistance (SAR) and induced-systemic resistance (ISR) in distal tissue.

Effectors are molecules that are secreted from pathogens or beneficial microorganisms and affect the outcome of their interaction with their host, such as participating in successful colonization or pathogenicity processes (Figure 1 and Figure 2; Table 1). Most of the plant pathogen-secreted effectors function inside the host plant cell and are mostly virulence-promoting factors that inhibit or interfere with the plant’s defense system [72,73,74,75]. A few recent studies have suggested that effectors may also contribute to the BCAs’ ability to control plant pathogens [76,77,78,79]. In *Trichoderma* spp., some effectors have been shown to be involved in antagonistic effects, such as the cerato-platanin protein Sm1 and its orthologue EPl1 that induce ROS production and pathogenesis-related (PR) gene expression in the host plant [80,81,82]; (For review see Ramírez-Valdespino et al., 2019 [79]). Two other *Trichoderma* proteins, Sm2 and Epl2, were found to be more relevant to defense activation in the plant host, but their functions are unknown [83,84]. A recent study on *Trichoderma* spp. identified 16 effector-like proteins during its interaction with *Arabidopsis thaliana* and the plant pathogen *R. solani*. The transcription levels of those putative effectors were modified in response to either the host plant or the fungal pathogen, and some had predicted functions, such as proteases, LysM and thioredoxins. One of those genes, *Hydii1* from *T. virens*, was identified as a hydrophobin participating in root colonization and its expression was correlated with the enhanced antagonistic effect of *T. virens* against *R. solani* [78]. Other hydrophobins from *Trichoderma*, such as HBF2-6, Hytlo1 and Hyd1, were found to be involved in colonization and the host plant’s defense response. A study on the beneficial *F. oxysporum* CS-20 demonstrated recently that CS20EP a small cysteine-rich protein, predicted as secreted effector, elicits ion exchange and defense response against pathogenic *F. oxysporum* in tomato plants [85]. Studies comparing the genomes and secretomes of the pathogen *U. maydis* and other smut pathogens with the BCA *P. flocculosa* demonstrated that these fungi, which have very different lifestyles, share genome features, including hallmarks of pathogenicity [59,86]. This comprehensive work demonstrated that *P. flocculosa* had lost a specific subset of candidate secreted effectors connected to virulence but had acquired genes encoding secreted proteins believed to have direct relevance to its biocontrol ability against plant pathogens [59]. Another comparative study of four genomes of *Pseudozyma* species with that of *U. maydis* found 113 putative secreted effectors in common: some were validated as effectors, such as the Pep1 protein which suppresses microbe-associated molecular pattern (MAMP)-triggered ROS production [87]. An orthologue of Pep1 from *Pseudozyma* could also complement a Pep1-deficient *U. maydis* mutant, restoring its virulence [64]. These new subsets of secreted effectors may function as virulence factors against plant pathogens, as we recently demonstrated that a *P. aphidis*-secreted fraction can activate ROS production and PCD in *B. cinerea* hyphal cells [44]. A later study demonstrated hyperbiotrophy as a new mode of action, when a unique effector of *P. flocculosa* directed inside *Blumeria graminis* (powdery mildew) caused a flow of nutrients into *P. flocculosa* [77]. This disruption in the nutrient flux caused collapse of the powdery mildew haustoria, and the plant eventually recovered from the pathogen [65].

## 4. Induced Resistance by Fungal Metabolites

To cope with constant attacks by invading pathogens, plants have evolved a wide range of defense mechanisms, including the use of preexisting physical (e.g., cuticle and cell wall) and chemical (e.g., antimicrobial compounds) barriers [88]. Pathogens and beneficial microbes can be detected by their MAMPs, which are recognized by specific pattern-recognition receptors (PRRs). MAMP–PRR interactions can lead to a weak response termed pattern or MAMP-triggered immunity (Figure 2) [89]. Pathogens that pass these first layers of defense may encounter the hypersensitive response—the rapid induction of localized cell death—which prevents their spread beyond local infection [90,91]. Those pathogens that persist and overcome the hypersensitive response still need to challenge the well-orchestrated plant defense responses, which involve effector-triggered immunity that includes systemic acquired resistance (SAR)(Figure 2) [90,92,93,94]. SAR is a form of induced resistance in plants with a specific defense-signaling pathway that occurs systemically after localized exposure to a pathogen, and is especially useful against biotrophic pathogens. It is characterized by the accumulation of salicylic acid (SA) and the expression of SAR-marker PR genes [95], but it can also be SA-independent [96]. Some microorganisms are associated with induced systemic resistance (ISR), which does not involve the accumulation of SA. Instead, it is mediated by jasmonic acid (JA) and ethylene (ET), which co-regulate a set of defense-related genes, different from those regulated by SA [97,98]. The JA/ET defense-signaling pathway plays an important role in plant defense responses against necrotrophic pathogens and herbivorous insects [97,98,99].

Some beneficial microbes, especially growth-promoting bacteria and fungi in the rhizosphere, can enhance the induced resistance responses in plants, mainly via secreted elicitors (Figure 2; Table 1). While beneficial microbes are generally associated with ISR, they can also activate SAR. The specifically activated pathways may involve JA/ET, SA, or both [43,100,101]. Prolonged activation of inducible defense involves major costs that affect plant growth and reproduction. Therefore, for the SAR/ISR to be affordable and beneficial, it should mainly be activated when the plant is exposed to attack [94,102,103]. To achieve this, beneficial microbes induce a unique physiological state called priming, which causes the plant to respond with faster and stronger activation of the induced defense responses, but only following an attack by pathogens [104].

Several fungal BCAs, mainly of the genus *Trichoderma,* have been reported to elicit ISR and priming in colonized plants. For example, *T. harzianum* T39 primed grapevines against downy mildew [105]; and *T. asperellum* SKT-1 induced resistance in *Arabidopsis* against the bacterial pathogen *P. syringae* pv. *tomato* [106] and modulated the expression of genes involved in the JA/ET-signaling pathways of ISR [107]. The endophytic basidiomycete fungus *Piriformospora indica* primes various plant species against powdery mildew [108], and the protective *Fusarium oxysporum* strain Fo47 primes tomato plants against pathogenic *F. oxysporum* strains [109]. In addition to the direct control of phytopathogens, *P. aphidis* and *P. churashimaensis* were found to indirectly protect plants by local and systemic induction of SAR and ISR, and priming the plant’s defense machinery for stronger induced defense activation after pathogen infection [43,110]. However, *P. aphidis* suppressed MAMP-triggered callose formation on the plant leaf surface, suggesting that it bypasses the MAMP-triggered immunity to become established on the leaf surface, and only later induces plant resistance and priming (Figure 2; unpublished data).

Various chemicals have been discovered that seem to act at various points in the defense-activating networks and mimic all or part of the biological activation of resistance [111]. Two prominent examples of such chemicals are acibenzolar-s-methyl (ASM) and β-aminobutyric acid (BABA). ASM is a functional analogue of SA that acts downstream of SA accumulation through the activation of SA-response mechanisms. Application of this compound induces similar responses in plants to those induced by biotrophic pathogens or SA, including overexpression of PR genes [112], and it has been widely reported to induce resistance against a broad spectrum of pathogens in many plant species. BABA is a non-protein amino acid known to induce resistance against many plant pathogens in a range of crop plants [113] by both SA-dependent and SA-independent defense mechanisms [114].

Many natural compounds have also been claimed to elicit ISR and priming, including oligosaccharides, glycosides, amides, vitamins, carboxylic acids, and aromatic compounds. Most mimic pathogen interactions and are able to induce or prime defense in a concentration-dependent fashion. However, the mode of action of these elicitors is eventually determined by the hosts and the stress that they are facing [115,116,117,118]. Extracts of plants and microorganisms are a potential source for the discovery and isolation of new natural elicitors, such as hexanoic acid which is a natural compound produced by strawberry. This potent natural priming inducer can activate broad-spectrum defenses by inducing callose deposition and the SA and JA pathways [115,119]. While natural elicitors can occasionally be found anywhere in the natural world, as evidenced by the case of hexanoic acid, a more likely source of such compounds is fungal BCAs. Since the elicitation of ISR and priming by fungal BCAs naturally involves some kind of chemical mechanism, extracts of these fungi and their secretions are more likely to contain the natural elicitors, which can be isolated and identified. Hossain et al. [120] reported that root treatment with culture filtrate of the growth-promoting fungus *Penicillium simplicissimum* induced resistance to *P. syringae* pv. *tomato* in *Arabidopsis* plants [120]. ISR activation against the same pathogen in *Arabidopsis* plants was also induced by blends of volatile compounds extracted from the growth-promoting fungi *Ampelomyces* spp. and *Cladosporium* spp. m-cresol and methyl benzoate were identified as the major active compounds in these blends [121].

As in many cases with fungal BCAs, most of the extensive studies have been performed with *Trichoderma* spp. Djonović et al. [80] identified and purified a small protein (designated Sm1) from *T. virens* secretions that triggered production of ROS and induced the expression of defense-related genes, both locally and systemically, in cotton. Pretreatment of cotton cotyledons with Sm1 also provided high levels of protection against the pathogen *Colletotrichum* sp. [80] (see also Section 3). *T. virens* also produces unique peptaibols with antibiotic properties (see also Section 2) [31], but it also has the ability to induce resistance. Cucumber plants co-cultivated with *T. virens* strains disrupted in their ability to produce these peptaibols showed a significantly reduced systemic resistance response to the leaf pathogen *P. syringae* pv. *lachrymans*, and reduced ability to produce phenolic compounds with inhibitory activity. On the other hand, two synthetic peptaibol isoforms (TvBI and TvBII) from *T. virens* induced systemic protection to leaf-pathogenic bacteria, induced antimicrobial compounds in cucumber cotyledons, and upregulated the expression of several defense-related genes [122].

The resistance induced by fungal BCAs can also be elicited by small secreted molecules such as secondary metabolites. Vinale et al. [123] demonstrated that pre-application of secondary metabolites isolated from the culture filtrate of several biocontrol strains of *Trichoderma* reduces the disease symptoms of *B. cinerea* on tomato plants, and induces overexpression of PR proteins [123]. We recently found that application of *P. aphidis* extract (a fraction that does not contain antibiotic activity) can induce PR genes in tomato plants and activate the plant defense system (Harris R and Levy M unpublished data).

## 5. Mycoparasitisim and Cell Wall-Degrading Enzymes

The secreted fraction of BCAs contains factors other than antibiotics and elicitors/effectors that might play a part in their ability to control pathogens. Some of these are cell wall-degrading enzymes and proteases which have been well documented as being part of the mycoparasitic mode of action (Figure 1; Table 1). As early as 1932, Weindling [124] described mycoparasitism of *R. solani* hyphae by the hyphae of *Trichoderma lignorum*, including coiling around the pathogen hyphae, and penetration and subsequent dissolution of the *R. solani* cytoplasm [30]. Mycoparasitism was demonstrated as one of the BCA’s direct modes of action that relies on the production of fungal cell wall-degrading enzymes such as chitinases, the latter having been shown to affect pathogenicity of germinating conidia of *B. cinerea* [125,126]. However, it has also been shown that some *Trichoderma* spp. isolates that have the potential to produce cell wall-degrading enzymes do not necessarily provide good biocontrol activity [127].

The antagonistic activity of *P. aphidis* against powdery mildew seems to be mainly parasitic. Scanning electron microscopy analysis revealed that *P. aphidis* dimorph from yeast-like to hypha-like and ecto-parasitizes the powdery mildew hypha by coiling around it [44]. Similar mycoparasitism occurs with *P. flocculosa* on conidia of cucumber powdery mildew [59]. In contrast, we have shown that to control gray mold disease caused by *B. cinerea, P. aphidis* remains as a yeast-like structure and thus does not coil around *B. cinerea* hyphae [44]. However, the *P. aphidis* cells could adhere to *B. cinerea* hyphae and secrete lytic enzymes and proteases when exposed to *B. cinerea* cell wall extract, suggesting that close proximity enhances the antibiotic activity and cell wall-degrading enzyme efficiency, thereby also supporting the parasitism dogma [62]. The dimorphic switch from hypha-like to yeast-like is a well-known phenomenon in fungal pathogens [128]. In *C. albicans*, this dimorphism is essential for pathogenicity [129,130]. This morphological transition is mainly determined by environmental cues and conditions, such as pH, temperature and nutrient availability [128,131]. For example, *C. albicans* and *Penicillium marneffei* are yeast-like at low pH, while at higher pH they transform into hypha-like [132,133]. *Pseudozyma* spp. also demonstrate dimorphism in response to different cues [43,44], and we also detected *P. aphidis* with a hypha-like structure at high pH, whereas it was yeast-like at low pH (unpublished data).

## 6. Competition for Space and Nutrients

Competition can occur between the BCA and the pathogen on plants for either space or nutrients; competition for both can hinder plant pathogens and control their ability to proliferate and infect plants. Similar to other BCAs, different *Trichoderma* strains use divergent modes of action to control phytopathogens, including competition for space and for micro- or macronutrients [118]. Low availability or limited sources of these nutrients challenge BCAs to develop different strategies to utilize them. One strategy might be competition for space, ultimately excluding the pathogen from the host plant. For example, *T. harzianum* is able to control *B. cinerea* on grapes by colonizing the blossom tissue to compete for space and eventually excluding the pathogen from its infection site [134]. Other strategies rely on the ability of BCAs to mobilize nutrients using low pH [123], specialized enzymes such as invertase [135], sugar transporters [136,137] or siderophores [138]. The ability to use diverse carbon or nitrogen sources from the plant and the pathogen can also contribute to the competitive ability. For example, *Kloeckera apiculate* competing with *Penicillium italicum* for nutrients and vitamins has a lower spectrum of carbon sources but a wide range of nitrogen sources as compared to the pathogen [139]. There are some examples of combined competition for both space and nutrients, such as the BCA *Pichia guilliermondii* which controls *Rhizopus nigricans* on tomato fruit during storage [140]. *Aureobasidium pullulans* also controls the decay of apple fruit caused by *B. cinerea* and *Penicillium expansum* by competing for both space and nutrients [141]. Competition for space and nutrients was also demonstrated after application of *P. aphidis* on tomato plants. *P. aphidis* accumulated and proliferated significantly more in the lesion area of *B. cinerea* than in the uninfected area, suggesting competition for space [43]. Furthermore, using excess sugar on tomato fruit reduces the competition for nutrients between the fungi and thus decreases the efficacy of *P. aphidis* against *B. cinerea* [62]. The ability to utilize more sources of carbon or nitrogen will give the BCA the advantage over the pathogen, and this will eventually reduce the infection.

## 7. Conclusions

Health and environmental concerns, along with the rapid development of pathogen resistance to widely used pesticides, call for enhancing the use of BCAs and biopesticides developed from their secretions in the long term, for the benefit of the consumer and the growers. Most biocontrol modes of action are mediated by their secretion-based fraction, which can include metabolites and proteins with different functions—from antibiotics through effectors—and enzymes that can affect or interact with the pathogen and/or the plant host. Much attention is being given to the characterization of such BCA-secreted molecules and proteins, but the development of robust molecular research tools is needed. Recent work shows that *Pseudozyma* can be modified using CRISPR-Cas9 system [142] for gene editing, and being yeast-like, it is comparatively easier to transform than filamentous fungi [44]. Large-scale studies, such as tritrophic interactions and omics [77,143], albeit challenging, will also help decipher pathways and reveal novel targets in pathogens and plants for directed control mechanisms. These tools will help further elucidate the molecular mechanism underlying the BCAs’ secretion components and will advance our ability to improve BCAs’ modes of action to suppress plant pathogens.

Note: *P. antarctica, P. aphidis* and were recently transferred to the genera *Moesziomyces* [144] and *P. flocculosa* to *Anthracocystis* [145]. For this communication, we kept their original names as they appear in the publications to which we refer.

## Figures and Tables

**Table 1:** Overview of biological control agents (BCAs) modes of action mechanisms.

Biocontrol Agent	Compound/Protein/Gene	Mechanism/Activity	Pathogen	Ref.
Antibiosis/ ROS/ PCD				
***Acremonium strictum***	Verlamelin	Unknown	*Erysiphe graminis f.* sp. *hordei* *Puccnia recondita* *Botrytis cinerea*	[5,70]
***Trichoderma virens Trichoderma spp.***	Peptaibols	Formation of pores in bilayer lipid membrane.	*B. cinerea* *Mucor mucedo*	[3,6,31,33,122]
***Trichoderma pseudokoningii***	Peptaibols	Induces metacaspase-independent apoptotic cell death.	*Fusarium oxysporum*	[35]
***T. virens***	Peptaibols	Induced resistance.	*Pseudomonas syringae*	[3,12,122]
***Pseudozyma aphidis***	Mostly lipophilic compounds.	ROS/PCD	*Podosphaera xanthii* *B. cinerea* *Clavibacter michiganensis*	[44,61,62,101]
**Biocontrol Agent**	**Compound/Protein/Gene**	**Mechanism/Activity**	**Pathogen**	**Ref.**
***Psudozyma rugulosa*** ***Pseudozyma flocculosa* (syn.*Sporothrix flocculosa*) **	(Z)-9-heptadecenoic Z)-6-methyl-9-hepta decenoic acids Cis-9-Heptadecenoic acid (CHDA)	Disturbance in fluidity of the cell membrane, leakage of electrolytes and proteins.	*E. graminis* *var. tritici,* powdery mildews	[45,46,47,48,49,50]
***P. flocculosa***	Flocculosin	Leakage of cell membrane.	*Candida albicans,* *Trichosporon asahii,* powdery mildews	[53,54,55]
***Pseudozyma tsukubaensis*** ***Pseudozyma prolifica***	Mycocins	Membrane disruption.	Ustilaginomycetes	[51,52]
***Pseudozyma graminicola*** ***Pseudozyma fusiformata***	Ustilagic acid	Disruption of the cytoplasmic membrane permeability.	~300 tested species of yeastlike and mycelial fungi	[56,57]
***Chaetomium globosum***	Chaetoviridins A and B	Antibiosis	*Puccinia recondita, Magnaporthe grisea*	[67,68]
***Fusarium semitectum***	Fusapyrone and deoxyfusapyrone	Antibiosis	*B.cinerea,* *Aspergillus parasiticus,* and *Penicillium brevi*	[69]
***Gliocladium virens* p **	Gliovirin and heptelidic acid	Suppressing TNF-alpha synthesis.	*Pythium ultimum*	[30]
***G. irens* q **	Gliotoxin and dimethylgliotoxin	Oxidative stress	*Rhizoctonia solani*	[30]
**Effectors**				
***P. flocculosa***	pf02826, pf00303 and pf02382	Dissemination and sequestration of nutrient.	*Blumeria graminis*	[77]
***Pseudozyma* sp. **	Pep1, Cmu1, Cwh41 and Hum3	BCA -Plant interactions		[76,87]
***T. virens***	tvlysm1	Colonization of BCA and defense against the pathogen.	*Rhizoctonia solani*	[78]
***T. virens***	Cerato-platanin protein Sm1	Induces ROS and PR genes expression in cotton.	*Cochliobolus heterostrophus*	[80]
***T. virens***	Sm2 and Epl2	Induced resistance	*C. heterostrophus*	[80]
***Fusarium oxysporum* strain CS-20 **	CS20EP	Elicits defense responses and ion exchange in tomato plants.	*F. oxysporum*	[85]
**Hydrolytic Enzymes**				
***Trichoderma harzianum***	Endo-chitinase, chitobiosidase		*B.cinerea;* *F. oxysporum Sclerotium rolfsii*	[125,126,127]
***P. aphidis***	Chitinase, protease lipase, cellulase		*P. xanthii,* *B. cinerea*	[44,62]
**Parasitism**				
***Trichoderma lignorum***		Mycoparasitism, haustoria formation.	*R. solani*	[30,124]
***P. flocculosa***		Mycoparasitism, Hyperbiotrophy.	*P. xanthii* *B. graminis*	[55,77]
***P. aphidis***		Ecto-parasitism	*P. xanthii*	[44]
**Biocontrol Agent**	**Compound/Protein/Gene**	**Mechanism/Activity**	**Pathogen**	**Ref.**
**Induced resistance (IR)**				
***T. harzianum* T39 **	Through jasmonic acid(JA) and ethylene(ET) signals.	Induced resistance in grapevine.	*Plasmopara viticola*	[105]
***Trichoderma asperellum* SKT-1 **	Through salicylic acid (SA), JA and ET signaling pathways.	Induced resistance in *Arabidopsis thaliana*.	*P. syringae*	[106]
***T. asperellum* T203 **	Through JA and ET signals.	Induced resistance in cucumber.	*P. syringae*	[107]
***T. virens***	Sm1 and 18 mer peptaibols.	Induced resistance in cucumber.	*P. syringae*	[80,122]
***Piriformospora indica***	ISR, upregulation of PR genes and heat-shock proteins.	Induced resistance in barley.	*B. graminis*	[108]
***F. oxysporum* Fo47 **	Root colonization upregulation of GLUA and PR-1a.	Induced resistance in tomato.	Fusarium wilt	[109]
***Penicillium simplicissimum* GP17-2 **	Through SA, JA and ET signaling pathways.	Induced resistance in *A. thaliana.*	*P. syringae*	[111]
***Ampelomyces* sp. and *Cladosporium* sp**	Volatiles; *m*-cresol and methyl benzoate induce ISR.	Induced resistance in *A. thaliana.*	*P.syringae* pv. *tomato* DC3000	[121]
***P. aphidis***	Induce ISR and SAR through JA/ET and SA pathways.	Induce resistance in *A. thaliana*, tomato and cucumber.	*B. cinerea* *C. michiganensis*	[43,61,101]
**Competition**				
***T. harzianum***		Competition for space.	*B. cinerea*	[134]
***P. aphidis***		Competition for space and nutrients.	*P. xanthii,* *B. cinerea*	[43,44,62]
***Pichia guilliermondii***		Competition for space and nutrients.	*Rhizopus nigricans*	[140]
***Kloeckera apiculate***		Competition for nutrients	*Penicillium italicum*	[139]

Programmed cell Death, PCD; Reactive Oxygen Species, ROS; Tumor Necrosis Factor, TNF; Induced Systemic Resistance, ISR; Systemic Acquired Resistance, SAR.
